# Reproducible untargeted metabolomics workflow for exhaustive MS2 data acquisition of MS1 features

**DOI:** 10.1186/s13321-022-00586-8

**Published:** 2022-02-16

**Authors:** Miao Yu, Georgia Dolios, Lauren Petrick

**Affiliations:** 1 Department of Environmental Medicine and Public Health, Icahn School of Medicine at Mount Sinai, New York, NY 10029 USA; 2 The Institute for Exposomic Research, Icahn School of Medicine at Mount Sinai, New York, NY 10029 USA

**Keywords:** Metabolomics, High-resolution mass spectrometry, Reproducible research, Workflow, Data analysis, Open science

## Abstract

Unknown features in untargeted metabolomics and non-targeted analysis (NTA) are identified using fragment ions from MS/MS spectra to predict the structures of the unknown compounds. The precursor ion selected for fragmentation is commonly performed using data dependent acquisition (DDA) strategies or following statistical analysis using targeted MS/MS approaches. However, the selected precursor ions from DDA only cover a biased subset of the peaks or features found in full scan data. In addition, different statistical analysis can select different precursor ions for MS/MS analysis, which make the *post-hoc* validation of ions selected following a secondary analysis impossible for precursor ions selected by the original statistical method. Here we propose an automated, exhaustive, statistical model-free workflow: paired mass distance-dependent analysis (PMDDA), for reproducible untargeted mass spectrometry MS2 fragment ion collection of unknown compounds found in MS1 full scan. Our workflow first removes redundant peaks from MS1 data and then exports a list of precursor ions for pseudo-targeted MS/MS analysis on independent peaks. This workflow provides comprehensive coverage of MS2 collection on unknown compounds found in full scan analysis using a “one peak for one compound” workflow without a priori redundant peak information. We compared pseudo-spectra formation and the number of MS2 spectra linked to MS1 data using the PMDDA workflow to that obtained using CAMERA and RAMclustR algorithms. More annotated compounds, molecular networks, and unique MS/MS spectra were found using PMDDA compared with CAMERA and RAMClustR. In addition, PMDDA can generate a preferred ion list for iterative DDA to enhance coverage of compounds when instruments support such functions. Finally, compounds with signals in both positive and negative modes can be identified by the PMDDA workflow, to further reduce redundancies. The whole workflow is fully reproducible as a docker image xcmsrocker with both the original data and the data processing template.

**Graphical Abstract:**

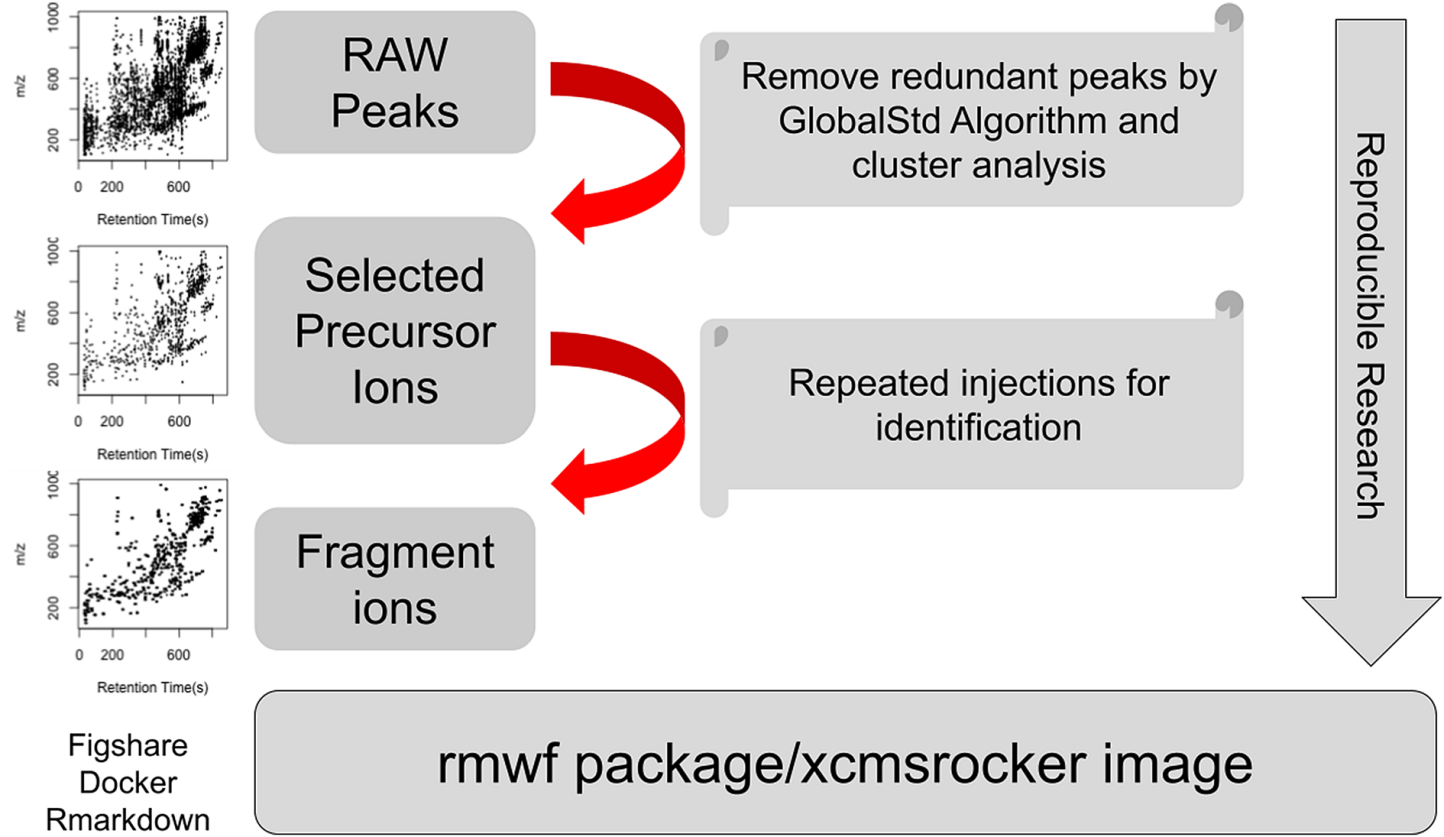

**Supplementary Information:**

The online version contains supplementary material available at 10.1186/s13321-022-00586-8.

## Introduction

Metabolomics often aims at revealing changes in levels of all possible metabolites in biological samples [1] and non-targeted analysis (NTA) usually aims at comprehensive profiling of compounds in environmental samples [2]. To achieve these goals, both approaches use high-resolution mass spectrometry (HRMS) to perform unbiased measurement of small molecules followed by identification of unknowns [3]. In most HRMS-based workflows, small molecule profiles will first be extracted across samples as peaks or features [4]. Tens of thousands of features are typically extracted in each sample, making it impractical to target every feature for MS/MS fragmentation [5]. For biological studies comparing subject groups, statistical analysis, machine learning algorithms, and annotation of isotopes, adducts, and neutral losses can be performed to subset the features into peaks of interest [6, 7]. Those selected peaks are then targeted for MS/MS fragmentation for identification. However, this approach is limited to a single research question and statistical analysis, as a secondary question or analysis would reveal different ions as targets for MS/MS analysis which may not be possible to acquire years after the original data acquisition [8]. In contrast, group comparisons may not be available in ecological study designs or environmental investigations for supervised statistical analysis [9]. In this case, an exhaustive MS2 collection strategy of all possible small molecules with reliable MS1 measures needs to be developed to maximize potential metabolite annotations, as well as increasing the reproducibility between the MS1 measurements and MS2 acquisition.

Automated untargeted MS/MS identification techniques such as data-independent acquisition (DIA) and data dependent acquisition (DDA) are powerful tools in qualitative untargeted analysis for identification of unknowns [10]. For DDA, precursor ions for MS/MS are selected during data collection by user-defined strategies. For DIA, all ions are sent into the collision cell for fragmentation, and deconvolution algorithms are used to connect the fragment ions to the parent compounds. However, DDA and DIA cover only a subset of the full scan features and the selected precursor ions may come from background instead of biologically relevant features [11]. In addition, DDA and DIA are designed for qualitative analysis instead of performing quantitative or semiquantitative analysis with fragment ions [12], because a compromise must be made between more scan time for high quality fragment ions and well-shaped chromatography for precursor ions. Proposed solutions include time-staggered precursor ion lists as inclusion lists [13] or automated exclusion lists to cover more compounds during repeated DDA injections [14]. A more extensive preferred list of precursor ions can extend the coverage of DDA [15].

As an alternative to DDA or DIA, targeted MS/MS is a straightforward method for qualitative, semiquantitative, and quantitative analysis of known compounds. Since targeted MS/MS analysis requires a pre-defined peak list for both precursor and fragment ions [13], new strategies needed to be developed for implementation in untargeted analysis for discovery and hypothesis generation. Mainly, since redundant peaks dominate full scan mass spectra, targeted MS/MS peak lists need to be refined by pseudo-spectra annotation, i.e., clustering all mass spectral signals stemming from each metabolite [7, 16]. In practice, the number of unique compounds may be as little as twenty percent of the total feature numbers [17]. If only a single peak is selected as the precursor ion for each unknown compound, the numbers of precursors for targeted MS/MS are drastically reduced and opportunities for more extensive MS/MS compound coverage emerge.

Such a “one feature for one compound” strategy has been reported for several metabolomics studies [18, 19], mainly using known adducts, neutral loss, and isotope patterns to detect the redundant peaks. Software packages such as CAMERA [20] and RamClustR [21] have been developed to annotate the pseudo-spectra for unknown full scan mass spectra algorithms that use correlation of peaks and pre-defined paired-mass distances for selecting redundant peaks to generate pseudo-spectra [7]. However, adducts or in-source reactions might be quite different among different sample matrices or instrument parameters [22], even for peaks from the same compound [23]. Therefore, a frequency-based paired-mass distances algorithm, such as the GlobalStd algorithm, could be an alternative solution to determine pseudo-spectra for exhaustive and local MS/MS analysis as it is designed to extract independent peaks without predefined redundant peaks information [3, 17]. For example, sodium adducts should be considered only if paired mass distance (PMD) 21.98 Da appears in high frequency. Some of the high frequency PMDs belong to known adducts while others might belong to unknown adducts, oligomers or combinations of known adducts. GlobalStd is designed to remove the study-specific redundant peaks instead of using predefined adducts or reaction lists.

The reproducibility between the MS1 measurements and MS2 acquisition is only part of the untargeted metabolomics workflow with such high complexity and no gold standard for metabolomics data pre-processing, overall reproducibility of the workflow needs more improvement. Though raw metabolomics data can be uploaded and accessed through online databases such as MetaboLights [24] or metabolomics workbench [25], details of data analysis are not always transparent, and reduce the ability to fully reproduce the reported findings [26]. Data analysis software with a graphic user interface (GUI) can be easy to use and document, but is also restricted to only defined operations [27]. An open source data processing script can represent every step of the data analysis while still being flexible [28], but researchers need to adopt specific software within an integrated development environment (IDE), which also reduces reproducibility due to the lack of experience with certain software [29]. To address these challenges, a system image with pre-installed open source software and data process templates for untargeted analysis should be developed to attain fully reproducible omics studies.

**Fig. 1:**
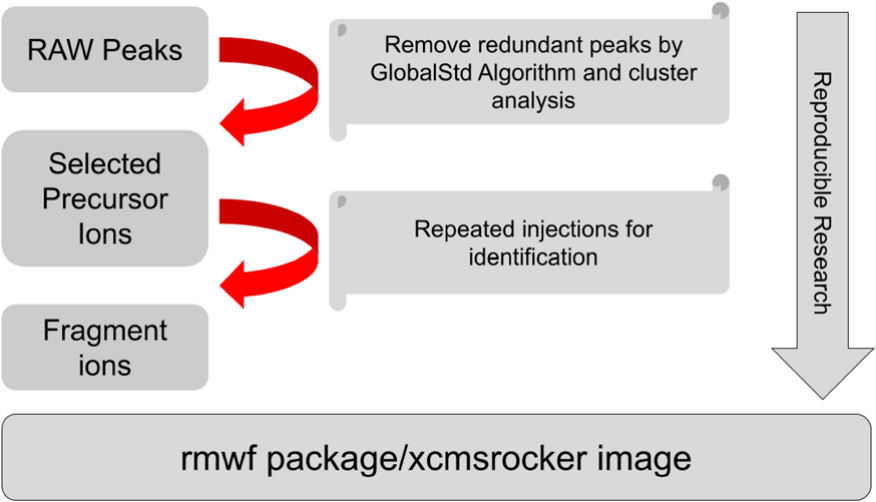
PMDDA workflow. Raw peaks are filtered by GlobalStd Algorithm to remove redundant peaks, then the remaining peaks are merged by cluster analysis to generate the precursor ion list. The selected peaks are assigned into multiple injections to collect the fragment ions for structure identification. The whole analysis can be found as a data process template in the ‘rmwf’ package. The complete data analysis is reproducible by xcmsrocker image

In this work, we developed a reproducible untargeted metabolomics data analysis workflow called paired-mass distance dependent analysis (PMDDA) which creates a study-specific list of independent peaks as precursor ions for MS/MS annotation based on MS1 full scan data (see Fig. 1). The purpose of this workflow is to increase the reproducibility between MS1 full scan and MS2 spectra by expanding the number of unique MS2 spectra collected that have precursor ions in the MS1 data. As a demonstration of the workflow we compared PMDDA with CAMERA and RamClustR precursor peaks selection algorithms using data acquired on standard reference material (NIST 1950, human plasma sample). We also integrated PMDDA selected precursor ions with iterative DDA as a preferred ions list to expand the coverage of MS/MS data collected on MS1 features. The utility of PMDDA was further demonstrated by finding the overlap in peaks between positive and negative mode analysis. All of the data and data processing scripts are also reproducible by a publicly available docker image, as well as a public GitHub repository.

## Results and discussion

### Precursor ion selection for MS/MS analysis

Using full scan mode, 6715 and 4666 features were measured in the NIST samples in positive and negative mode, respectively. After removal of peaks with fold change smaller than three times that of corresponding matrix samples and those peaks with a RSD larger than 30%, 4711 and 3608 features remained in positive and negative mode, respectively, as potential precursor ions for MS/MS analysis.

For PMDDA, the GlobalStd algorithm was used to reduce the redundant peaks [17]. To select precursors for targeted analysis, each reduced independent peak was linked to their paired high frequency PMD ions as an ion cluster, or pseudo-spectra. Clusters were merged if independent peaks could be linked to the same paired ions. In addition, since ions within clusters should be highly correlated, Pearson correlation coefficients smaller than 0.9 between paired mass distances were used as a threshold to exclude unrelated peaks from the same compounds. For each merged ion cluster, the peak with the highest intensity was selected as the precursor ion for MS/MS analysis. For the SRM samples, in positive mode, 849 independent peaks were selected by the GlobalStd algorithm, in which 780 precursor peaks were selected as annotations for targeted analysis after cluster analysis. In negative mode, 761 independent peaks generated 723 precursor peaks as annotations for targeted analysis. These annotations were also used as preferred ions using an iterative DDA strategy for comparison.

Precursor lists were also generated for CAMERA and RAMClustR using default settings. For CAMERA [20], peak cluster groups following annotation of the feature table were treated as pseudo-spectra, and the proposed molecular masses for each pseudo-spectra were extracted. Then, the [M + H]^+^ for positive mode and [M − H]^−^ for negative mode were generated as precursor ions for targeted analysis. For the SRM samples using CAMERA, 862 and 710 precursor ions were generated for MS/MS annotation for positive and negative mode, respectively. Similarly, RAMClustR [21] generated the molecular masses of each pseudo-spectra, and the corresponding molecular ions ([M + H]^+^ for positive mode and [M − H]^−^ for negative mode) were generated for targeted MS/MS analysis. For the SRM samples using RAMclustR, 542 and 770 precursor ions were generated for targeted analysis in positive and negative modes, respectively.

While several thousand features were measured in full-scan, pseudo-spectra generation by PMDDA, CAMERA, and RAMclustR resulted in less than 1000 unique features for MS/MS precursor ion selection, covering approximately 15% and 20% of the total feature numbers in positive and negative mode, respectively (see Additional file 1: Figs. S1 and S2). Nevertheless, obtaining high quality MS/MS spectra for all of those features in a single injection with high sensitivity is challenging. In this case, the precursor ions were randomly assigned into multiple injections to make sure that no more than 6 ions were scanned within a retention time shift of 0.2 min of the original retention time from full scan. Such repeated injections for PMDDA, CAMERA, and RAMClustR were aimed to retain high sensitivity and compound coverage, and could be implemented into untargeted studies using pooled QC samples for untargeted MS/MS analysis.

### Precursor selection comparison with CAMERA and RamClustR

The chemical coverage of different methods were compared based on molecular networks (spectra sets from related molecules, not necessarily matching to any known compounds) found by GNPS, as well as compound annotation results from only MS2 data on MS1 collected precursors. Here, only the molecular networking results that match with precursor ions found in MS1 full scan were kept for comparison, as only those results would be valuable to the analysis and interpretation of the study. We also included iDDA which utilizes an automatic iterative MS/MS collection from the preferred list of PMDDA precursor ions. Since the database-based annotation is biased towards compounds with available spectral data, and GNPS molecular networks may have multiple spectra from the same compounds, we also compared, by open source software xcms for MS2 spectra extraction, the number of unique MS1 compounds for which there was MS2 spectral data collected for CAMERA, RAMClustR, PMDDA. Since NIST 1950 samples contain known compounds, we also compared the results based on those results. Additional file 1: Fig. S1 and S2 visualized MS1 full scan peaks covered by MS2 precursor ions using different precursor selection methods.

**Fig. 2:**
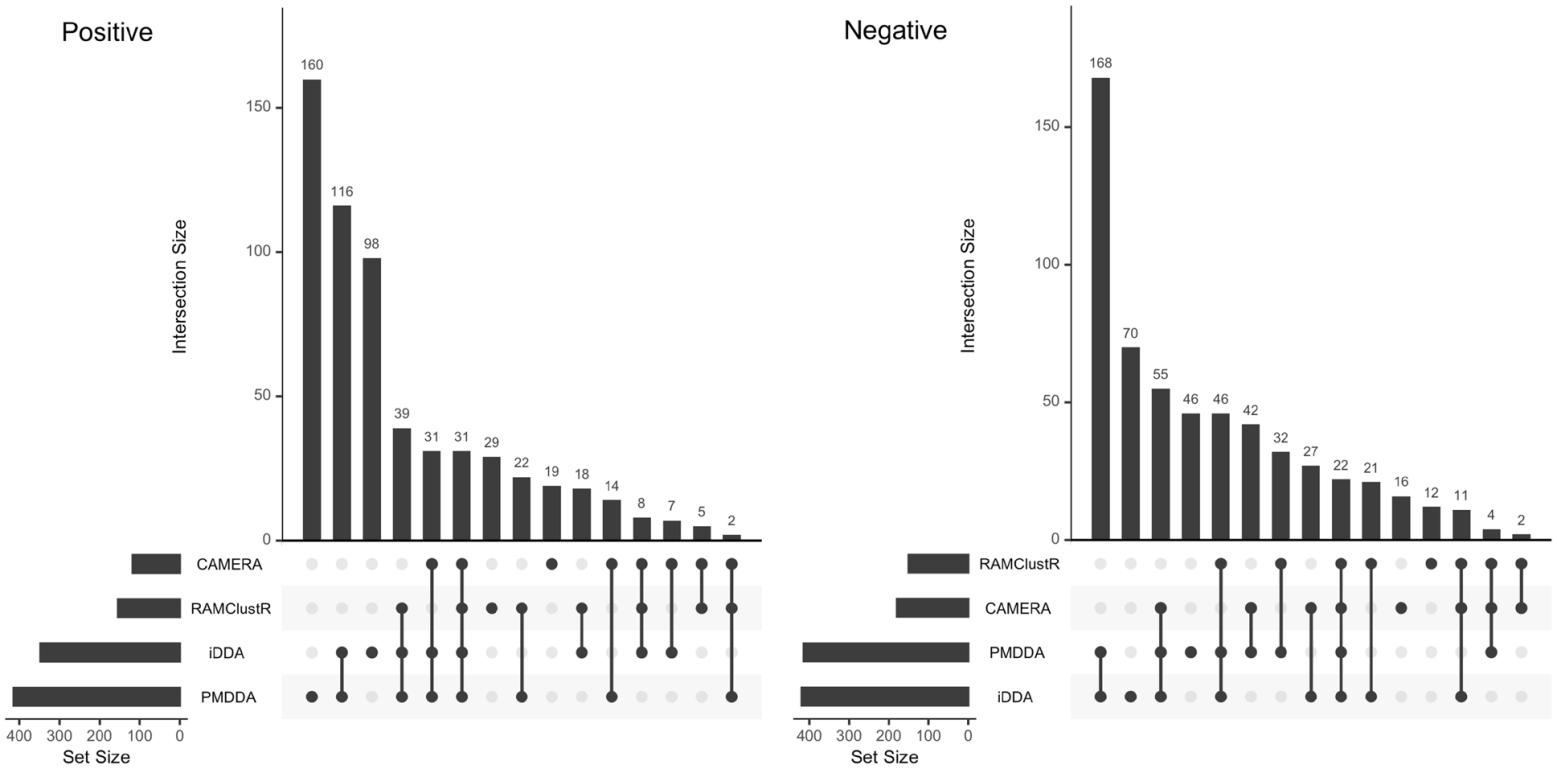
UpSet plot of metabolites networks found from CAMERA selected ions, RAMClustR selected ions, PMDDA selected ions, and iterative DDA (left panel is positive mode data and right panel is negative mode data). The set of ‘iDDA’ means iterative DDA with PMDDA selected precursor ions as the preferred list

For the molecular networking results from GNPS, as shown in Fig. 2, PMDDA found 160 unique molecular networks and iDDA found 98 unique molecular networks while they shared 116 unique molecular networks between them. Both CAMERA and RAMclustR found fewer unique molecular networks compared with PMDDA, 19 and 29, respectively. While RAMclustR and CAMERA shared 39 and 31 networks with both PMDDA and iDDA, only 31 molecular networks were found in all four methods. Interestingly, RAMclustR and CAMERA shared more molecular networks with PMDDA (22 and14, respectively) than iDDA (18 and 7, respectively). Results for negative mode were similar. As shown in Fig. 2, PMDDA found 46 unique molecular networks and iDDA found 70 unique molecular networks. PMDDA and iDDA shared 168 unique molecular networks. Both CAMERA and RAMclustR found fewer unique molecular networks compared with PMDDA with16 and 12, respectively. However, only 22 unique molecular networks were found in all four methods. In summary, PMDDA showed more molecular networks compared with RAMclustR and CAMERA.

We also compare different methods by the compound annotation results from GNPS. For positive mode, PMDDA found 73 compounds and iDDA found 77 compounds. Both CAMERA and RAMclustR annotated fewer compounds, 29 and 41, respectively. However, only 16 compounds were annotated in all four methods. PMDDA annotated 6 unique compounds with another 23 compounds were shared between PMDDA and iDDA. RAMClustR only annotated three unique compounds and no unique compounds were annotated with CAMERA. For negative mode, as shown in Additional file 1: Fig. S3, PMDDA annotated 36 unique compounds, iDDA found 45 unique compounds, CAMERA found 10 unique compounds, and RAMClustR found 16 unique compounds. PMDDA and iDDA shared 18 compounds while iDDA found 6 unique compounds. Interestingly, PMDDA did not annotate any unique compounds not shared by iDDA. Only 4 compounds were overlapping between PMDDA, iDDA, CAMERA, and RAMClustR. Both CAMERA and RAMclustR had no unique compounds found. In this case, PMDDA outperformed CAMERA and RAMclustR and it would be helpful to perform iDDA to extend the coverage of annotated compounds by GNPS.

As for the MS2 spectra extracted by xcms, PMDDA could extract 293 spectra for unique MS1 compounds, more than CAMERA (34) or RAMClustR (163) for positive mode. For negative mode, again, PMDDA found 254 spectra matching to unique MS1 data, more than CAMERA (46) and RAMClustR (150).

Known compounds in NIST 1950 were also compared among different methods. For positive mode, 6, 3 and 5 ions matched in PMDDA, CAMERA and RAMClustR’s precursor ions list while 12, 9 and 4 ions matched in negative mode, respectively. This suggests that PMDDA performs as well or better than the other precursor selection algorithms for selecting biologically relevant compounds for MS/MS annotation.

Overall, PMDDA showed better coverage than both CAMERA or RAMClustR for untargeted MS2 collection and annotation of metabolites measured in MS1 scan. This may be due to the fact that CAMERA and RAMClustR use pre-defined paired mass distances for adducts or redundant peaks, which may not accurately represent the specific sample type. PMDDA, on the other hand, employs a data-driven process (GlobalStd algorithm [17]) to find high frequency paired mass distances within the pseudo spectra, which may cover more unknown adducts or redundant peaks [17]. As shown in Additional file 1: Fig. S4 and S5, some of the high frequency PMDs belong to known adducts (e.g. 21.98 Da for sodium adducts, 18.01 Da for neutral loss of water) while others might belong to unknown adducts, oligomers or combinations of known adducts. Another difference between PMDDA, CAMERA, and RAMClustR is the software design. The pmd package is designed to remove redundant peaks while CAMERA and RAMClustR are designed for annotation directly from the feature peak table. As such, the latter algorithms have not been optimized for generating a precursor list for MS/MS which may have decreased performance compared to PMDDA.

When we include the results from iDD with the PMDDA selected precursor as the preferred list, the annotation performance can be further improved. However, PMDDA contains some unique annotations missing by iterative DDA (see Additional file 1: Fig. S3). On the other hand, as shown in Additional file 1: Fig. S6, iDDA can cover compounds with lower MS1 full scan intensity missing by other methods. A combination of PMDDA as preferred ions list and iDDA data collection should be considered to reach a larger coverage of peaks found in MS1 full scan when the hardware supports such data acquisition mode.

### Compounds identified in both negative and positive ionization modes

To expand metabolite coverage, the same sample is typically analyzed under both negative and positive electrospray ionization modes for a given chromatography and statistical analysis performed separately for both assays. However, compounds do not show the same ionization behavior in different modes, and respective peaks may be present in only one ionization mode or in both. This causes challenges for statistical analysis methods, such as false discovery rate control, which are highly dependent on the independent numbers of total compounds [30]. To overcome this, connections between negative and positive mode can be built after MS/MS annotation or identification, which might introduce bias on downstream statistical analysis. A previous study used correlation analysis to screen the same compounds in both modes [31], which can be influenced by redundant peaks from the same compounds. As an alternative, untargeted features present in both positive mode and negative mode can be determined using PMD.

**Fig. 3:**
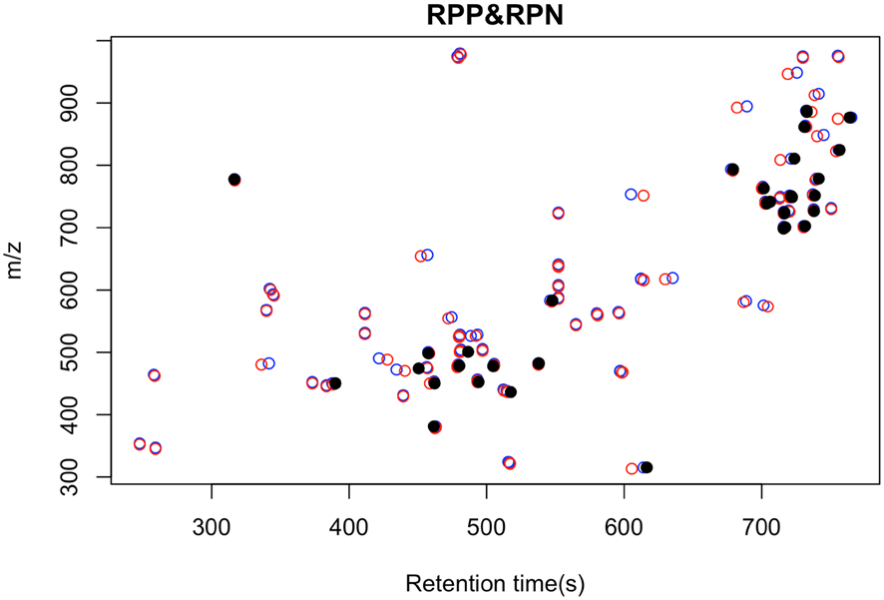
Features linked between positive and negative by PMD 2.02 Da within a retention time shift of 10 s for positive and negative mode ionization. The red and blue circles represent positive and negative ions, respectively. Compounds with confirmed identities based on MS/MS annotation to GNPS are colored in black

Untargeted features present in both positive and negative mode can be linked by paired mass distance of 2.02 Da representing the difference between [M + H]^+^ and [M − H]^−^ in the two modes. For SRM samples, we found 100 peaks that could be linked with 2.02 Da within a retention time shift of 10 s (see Fig. 3). MS/MS annotation of those 100 peaks using PMDDA identified 35 unique compounds with GNPS, only 2 of which had the same annotation [PE(P-16:0/20:4) and PE(P-16:0/18:2)] in both negative and positive mode, due to the absence of a library spectra in the opposite mode. Since spectral annotation databases might contain a more expansive coverage of only one ionization mode for certain compounds, linking through PMD could both reduce the potential redundant annotations and facilitate annotation of unknowns. By linking features in positive and negative mode, the total number of independent metabolites is reduced for choosing the appropriate downstream statistical analysis. A limitation of the current algorithm is that this linkage only works on data analyzed on the same chromatography column and gradient.

### Reproducible research

We aimed to maximize reproducibility of this research. Therefore, we used SRM samples that are commercially available and commonly used in metabolomics workflows, and made the raw data accessible online for future potential research purposes. In order to provide full transparency on the data analysis, we choose a command line based script within a graphic user interface to make sure every step is recorded and reproducible by other researchers [27]. A docker image, xcmsrocker was created based on Rocker image [32], which pre-installs most of the R-based metabolomics and NTA data analysis software. This docker image is available online and can be installed on any personal computer, workstation, or cloud computation platform with RStudio as IDE33. Software used for this workflow such as IPO, xcms, pmd, CAMERA, and RAMClustR had been pre-installed. The R package rmwf (https://github.com/yufree/rmwf) is also included with the data processing script of this PMDDA workflow as a template, as well as other workflow templates such as peak picking, annotation, or statistical analysis for different software. ‘xcmsrocker’ is freely available for download at https://hub.docker.com/r/yufree/xcmsrocker and source code on GitHub (https://github.com/yufree/xcmsrocker) [34].

## Conclusion

In this work, we propose an automated, reproducible, and exhaustive workflow to maximize MS2 collection on precursor ions selection from full scan mode untargeted metabolomics data. We demonstrated that PMDDA outperforms both CAMERA and RAMClustR for breadth of pseudo-spectra precursor ions selection. In addition, this workflow can be coupled with iterative DDA to cover more compounds found in MS1 full-scan. The PMDDA workflow was also able to identify features present in both negative and positive ionization modes, demonstrating additional utility of the workflow to reduce duplicates for downstream statistical analysis. The PMDDA workflow is fully open source, reproducible, and includes all raw data and data processing scripts available online.

## Methods

### Sample preparation

NIST 1950 Frozen Human Plasma standard reference material (SRM), which documented 85 compounds in the sample, was used in this study for reproducibility. Aliquots of 50 μL of NIST SRM plasma were thawed on ice. Proteins were precipitated by the addition of 150 μL of ice-cold methanol containing isotope labelled internal standards, 10 s of vortexing, and 30 min incubation at − 80 °C. The samples were then centrifuged at 13,000 g for 10 min at 4 °C, and 70 μL of the supernatant was transferred to two 1.5 mL microcentrifuge tubes. The extracts were evaporated using a Savant SpeedVac concentrator at 35 °C for 90 min and samples were stored at − 80 °C until analysis. Following the same protocol, 50 μL aliquots of a matrix blank (replacing the SRM plasma with water), were extracted.

### Instrument analysis

Immediately prior to data acquisition, dried samples were reconstituted in 60 μL of methanol. Samples were analyzed using an ultra-high performance liquid chromatography (UHPLC) 1290 Infinity II system (including 0.3 µm inline filter, Agilent Technologies, Santa Clara, USA) with 1260 Infinity II isocratic pump (including 1:100 splitter) coupled to a 6545 quadrupole-time time of flight (Q-TOF) mass spectrometer with a dual AJS electrospray ionization source (Agilent Technologies, Santa Clara, USA). Samples were maintained at 4 °C in the multisampler module. Reference masses included positive ionization mode: purine (m/z 121.0509), HP-0921 (m/z 922.0098); and negative ionization mode: purine (m/z 119.0363), HP-0921 (m/z 966.0007). Sheath and drying gas (Nitrogen purity > 99.999%) flows were 12 L/min and 10 L/min, respectively. Drying and sheath gas was 250 °C, with the nebulizer pressure at 20 psig, and voltages for positive and negative ionization modes at + 3000 V and − 3000 V, respectively.

The extracts were injected onto a Zorbax Eclipse Plus C18, RRHD column (50 mm × 2.1 mm, 1.8 µm particle size, Agilent Technologies, Santa Clara, USA) coupled to a guard column (5 mm × 2 mm, 1.8 µm Agilent Technologies, Santa Clara, USA) maintained at 50 °C. Separation occurred using Mobile phase A consisted of water with 0.1% formic acid and Mobile phase B consisted of 2-propanol:ACN (90:10, v/v) with 0.1% formic acid at a flow rate of 0.4 mL/min. A 15 min gradient was used (5% B for 2 min, increasing to 30% B in 2 min, and increasing from 30 to 98% B in 9.5 min with a 1.5 min hold), followed by a column re-equilibration phase. Data was acquired with a mass range of 100–1000 m/z (MS1) and 20–1000 m/z (MS/MS). The scan rate for MS1 full scan is 1.67spectra/s. The targeted analysis/ iterative DDA scan rate for MS1 is 4 spectra/s and 2 spectra/s for MS2 and 4 max precursors per cycle was set for iterative DDA.

Five SRM samples and five matrix blanks were analyzed. Data were collected in full scan positive and negative mode. Then, the precursor ions were selected for MS/MS fragmentation based on full scan data either via PMDDA, CAMERA, or RAMClustR. Peak lists for repeated injections of MS/MS analysis were automatically generated by an in-house script. The collision energy was set at 20 eV for all MS/MS fragmentation. In addition, eight injections of iterative DDA with PMDDA selected precursors as the preferred ions list were performed [15]. For iterative DDA, ions selected as precursors in previous injections are removed from the list in the following injections. Use of a preferred ions list ensures the selected ions were fragmented if they were in the samples.

### Data analysis

Data analysis was performed in R (version 4.0.2) [35] according to the workflow described in Fig. 1. Raw data were refined by retention time range between 30 and 930 s for the positive and negative mode to remove both the void volume and the washing phase of the column. The peak picking parameters for xcms [36] were optimized by IPO [37] for the five SRM samples. After retention time correction and peak filling for the low abundance peaks, the features were further filtered by those with intensity fold change larger than three times that in the SRM than the matrix samples. Peaks with relative standard deviation (RSD) larger than 30% in SRM samples were removed. The filtered peaks were linked with the MS2 annotation results from PMDDA, CAMERA, and RAMClustR selected precursor ions for comparison. Repeated injections were designed to retain high sensitivity for exhaustive MS/MS collection across the column gradient.

The MS/MS data were then converted to open source format (mzML) [38] and annotated using GNPS [39] molecular networking for MS/MS annotation with default settings (2 Da shift for precursor ions to include isotope and 0.5 Da shift for fragment ions). Then annotation results were linked back to MS1 full scan filtered data for further investigation with < 5 ppm shift of mass-to-charge ratio and < 5 s shift of retention time. Then the molecular networks and annotation results were compared among different methods.

SRM NIST 1950 contains 85 compounds with known exact masses including amino acids, fatty acids, clinical markers, etc. To compare the ability of each method to identify these known compounds, theoretical m/z for protonated and deprotonated ions were generated as [M + H]^+^ and [M − H]^−^ for positive and negative modes, respectively. Then, the precursor ions selected from PMDDA, CAMERA, and RAMClustR were aligned among the m/z ions list for these known compounds within two decimal places.

MS/MS spectra of the peaks matched to the filtered MS1 features list as MGF files were extracted for further investigation or improved matching to the algorithm/database. The MS2 spectra were extracted by combining spectrum from similar precursor ions within 0.02 Da, with fragment ions shifted < 5 ppm, and only including peaks that were larger than 60% of the remaining spectra.

The whole PMDDA workflow (Fig. 1), including MS1 feature extraction and filtering, precursor ion selection, and injection peak table generation for MS/MS analysis has been included in the rmwf package’s data processing template with links to download the original data via figshare [40]. Here, the MS/MS analysis can be targeted analysis with the selected precursor ions and/or various data-dependent acquisition modes with selected precursor ions as preferred ions when the instrument supports this feature. In addition, the workflow and corresponding software were packaged into a docker image called xcmsrocker (https://hub.docker.com/repository/docker/yufree/xcmsrocker). We also supplied the script and data files in a GitHub repository (https://github.com/yufree/pmdda) for this study in additional files with description including MS1 peaks list and MS2 MGF files for reproducible purposes.

## Supplementary Information

**Additional file 1: Figure S1.** showed MS1 selected ions from MS2 precursor ions and the corresponding peaks found in MS1 full scan for positive mode. **Figure S2.** showed MS1 selected ions from MS2 precursor ions and the corresponding peaks found in MS1 full scan for negative mode. **Figure S3.** showed UpSet plot of annotated compounds found from CAMERA selected ions, RAMClustR selected ions, PMDDA selected ions, and iterative DDA (iDDA). **Figure S4.** showed high frequency paired mass distances within pseudo spectra in positive mode and their distribution across MS1 data. **Figure S5.** showed high frequency paired mass distances within pseudo spectra and their distribution across MS1 data in negative mode. Figure S6 showed boxplot for the full scan peaks intensity of unique precursor ions found in each MS2 spectra from different methods on log scale.

## Data Availability

The datasets supporting the conclusions of this article are available in the figshare repository (https://figshare.com/projects/Reproducilble_Metabolomics_WorkFlow/59777). Raw data in mzML format for MS1 full scan data of 5 NIST 1950 samples and 5 matrix samples, as well as MS2 files from PMDDA precursor ions (7 for positive mode and 7 for negative mode), PMDDA for iterative DDA(8 for positive mode and 8 for negative mode), CAMERA precursor ions (8 for positive mode and 12 for negative mode), RAMClustR precursor ions(4 for positive mode and 5 for negative mode) and positive–negative connection ions(2 for positive mode and 2 for negative mode). Those data could be automatically downloaded in the GitHub script, as well as in the xcmsrocker image through rmwf package. To use the template outside the xcmsrocker image, install ‘rmarkdown’ and ‘rmwf’ package (from GitHub) and run the following code in R: rmarkdown::draft("test.Rmd", template = "PMDDA", package = "rmwf"). The annotation results supporting the conclusions of this article are available in the GNPS repository. GNPS Molecular Networking results can be accessed online for positive mode (https://gnps.ucsd.edu/ProteoSAFe/status.jsp?task=47bd0f0c102c42c09a879ac02c6c302f) and negative mode (https://gnps.ucsd.edu/ProteoSAFe/status.jsp?task=9ace8dc88efd48919efbbfc487379b78). The data processing script supporting the conclusions of this article are available in the GitHub repository (https://github.com/yufree/pmdda). This repository is for this manuscript with reproducible documents and all the necessary data as csv files or mgf files. The generated documents can be accessed online as a web page (https://yufree.github.io/pmdda/script.html). Check readme.md for details.
