# Supplementary material for: Reproducible untargeted metabolomics workflow for exhaustive MS2 data acquisition of MS1 features
Source: J Cheminform. 2022 Feb 16;14:6. doi: 10.1186/s13321-022-00586-8 (PMC8848943; doi:10.1186/s13321-022-00586-8)
Supplement: Supplementary file 1 — Additional file 1: Figure S1. showed MS1 selected ions from MS2 precursor ions and the corresponding peaks found in MS1 full scan for positive mode. Figure S2. showed MS1 selected ions from MS2 precursor ions and the corresponding peaks found in MS1 full scan for negative mode. Figure S3. showed UpSet plot of annotated compounds found from CAMERA selected ions, RAMClustR selected ions, PMDDA selected ions, and iterative DDA (iDDA). Figure S4. showed high frequency paired mass distances within pseudo spectra in positive mode and their distribution across MS1 data. Figure S5. showed high frequency paired mass distances within pseudo spectra and their distribution across MS1 data in negative mode. Figure S6 showed boxplot for the full scan peaks intensity of unique precursor ions found in each MS2 spectra from different methods on log scale. [file 13321_2022_586_MOESM1_ESM.docx]

Supporting information for

Reproducible untargeted metabolomics workflow for exhaustive MS2 data acquisition of MS1 features

Miao Yu^a*^, Georgia Dolios^a^, Lauren Petrick^a,b^

^a^ Department of Environmental Medicine and Public Health, Icahn School of Medicine at Mount Sinai, New York, NY, 10029, United States

^b^ The Institute for Exposomic Research, Icahn School of Medicine at Mount Sinai, NY, 10029, United States

*Corresponding author: Email: miao.yu@mssm.edu Phone: +1-646-707-5791. Fax: +1-646-537-9654.

**
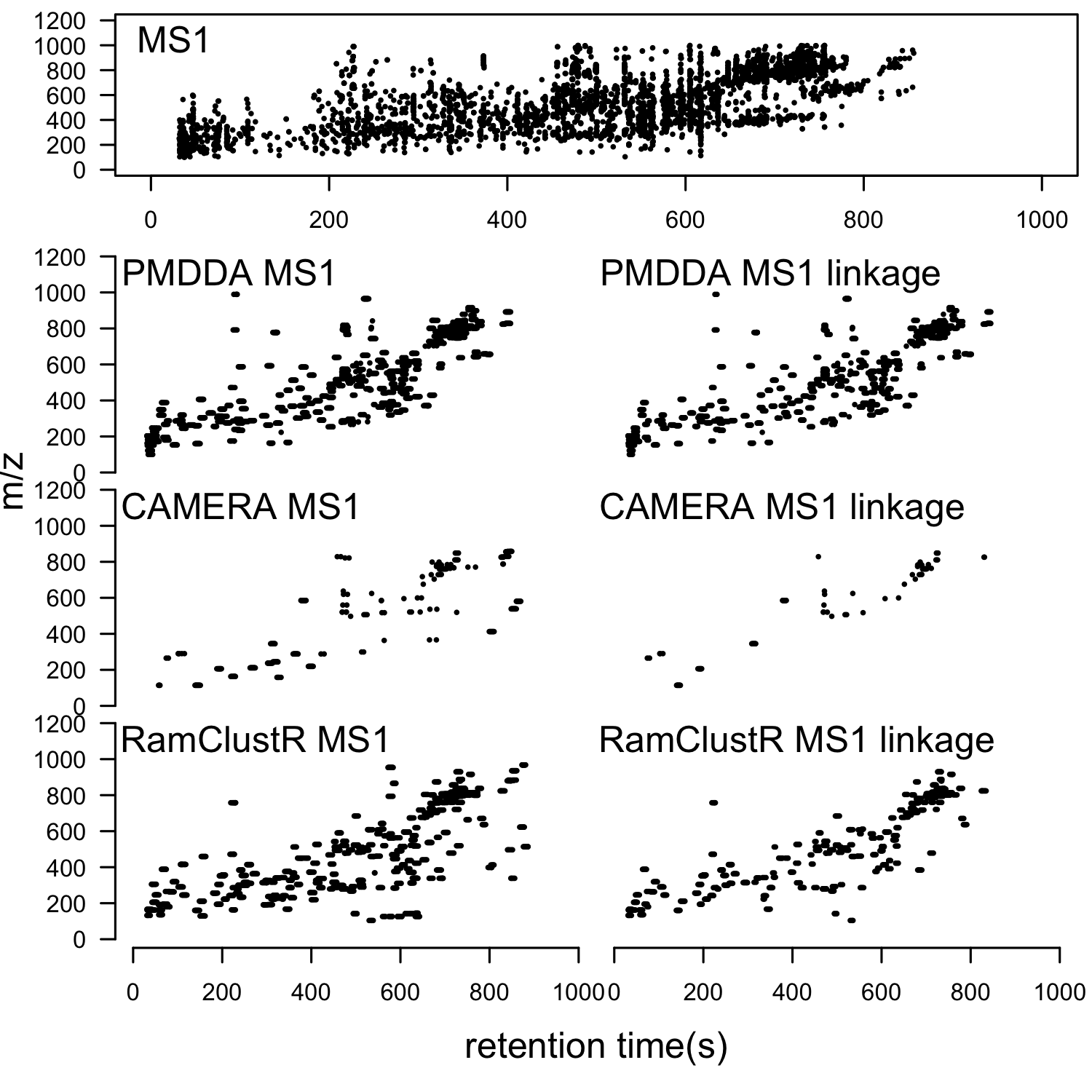
**

Figure S1. MS1 selected ions from MS2 precursor ions and the corresponding peaks found in MS1 full scan for positive mode.The upper panel shows the peaks from MS1 full scan and the lower panel show the precursor peaks collected in PMDDA/CAMERA/RAMClustR in the left and the linked peaks with MS 1 full scan in the right within m/z shift of 5 ppm and retention time shift of 5s.

**
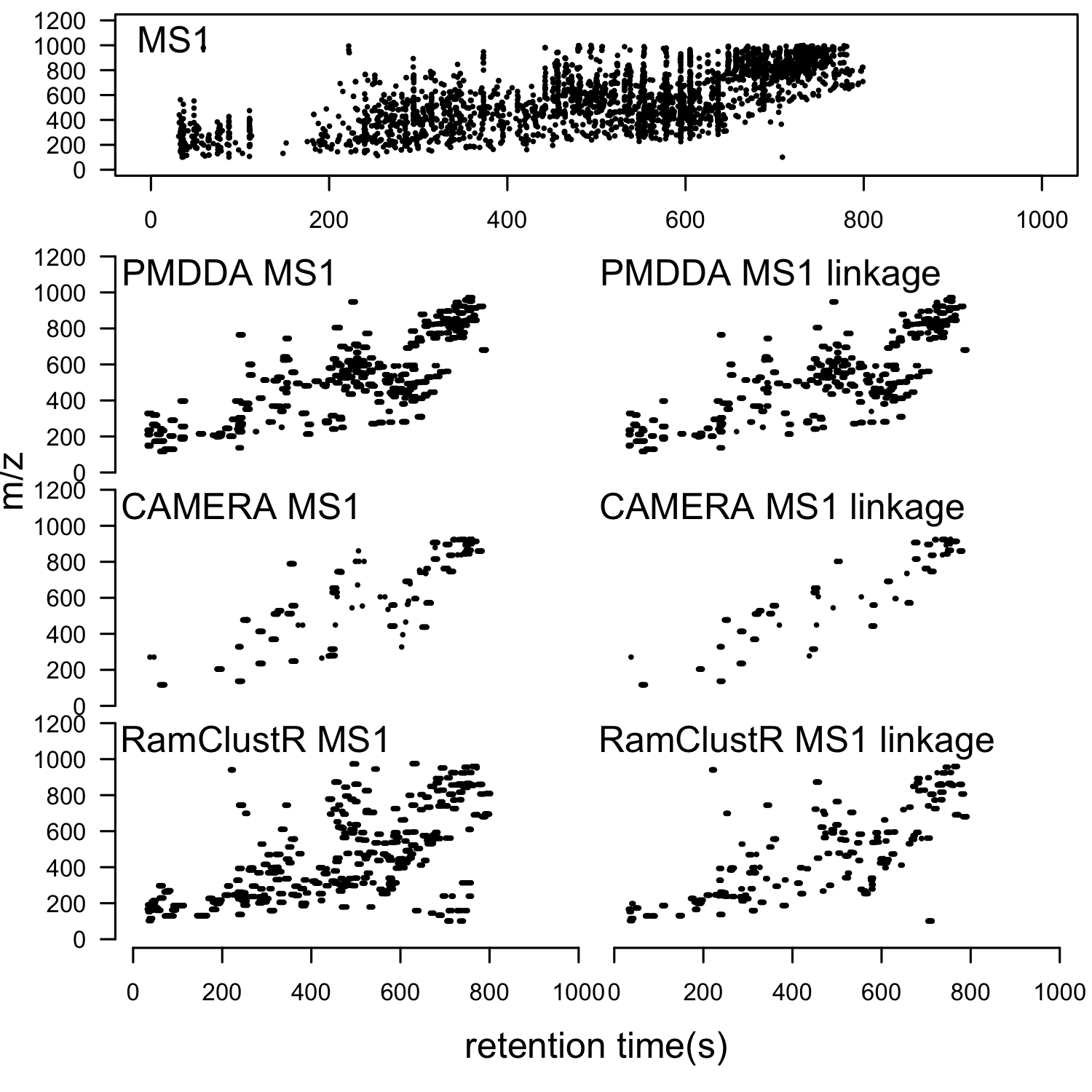
**

Figure S2. MS1 selected ions from MS2 precursor ions and the corresponding peaks found in MS1 full scan for negative mode.The upper panel shows the peaks from MS1 full scan and the lower panel show the precursor peaks collected in PMDDA/CAMERA/RAMClustR in the left and the linked peaks with MS 1 full scan in the right within m/z shift of 5 ppm and retention time shift of 5s.


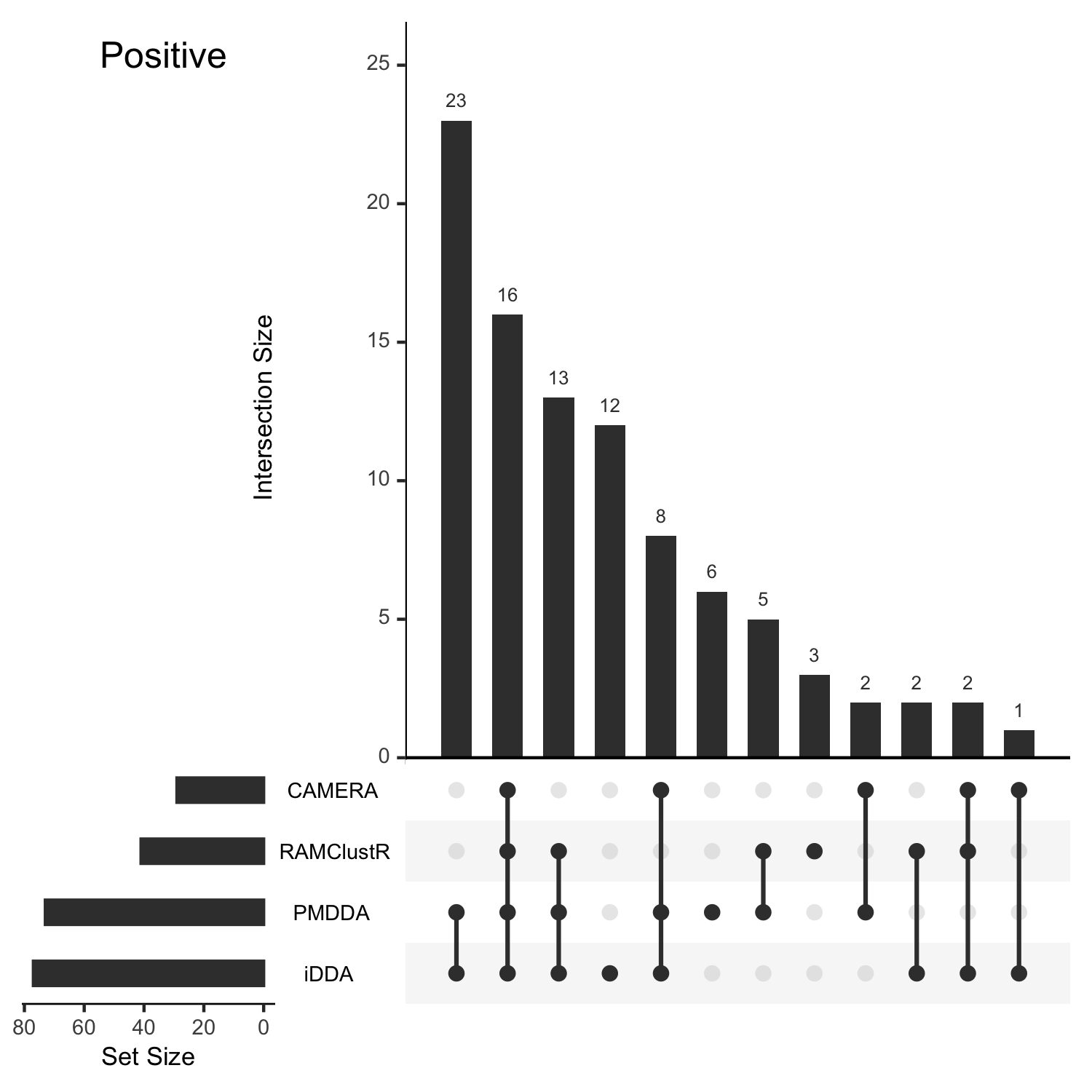

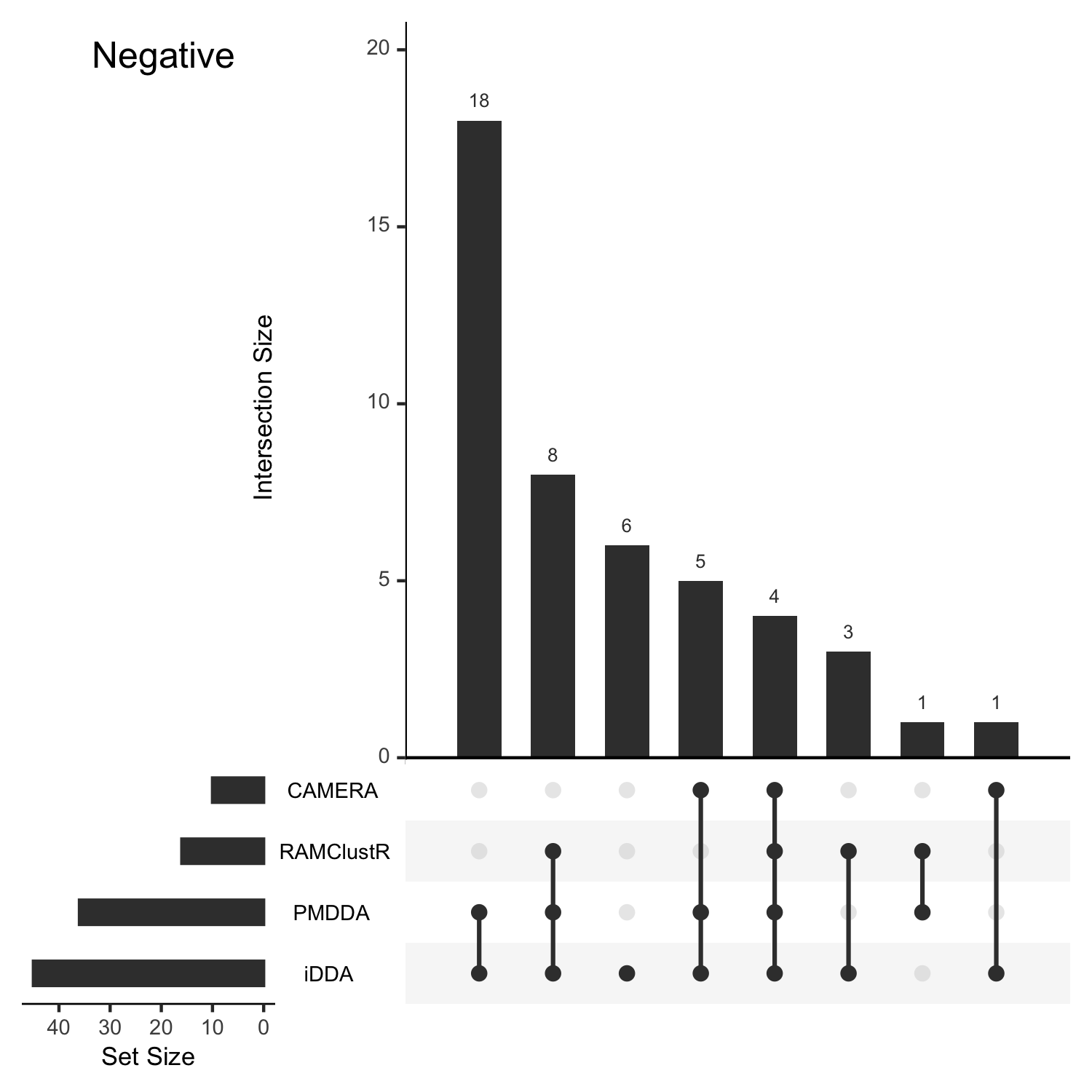


Figure S3. UpSet plot of annotated compounds found from CAMERA selected ions, RAMClustR selected ions, PMDDA selected ions, and iterative DDA (iDDA). The left panel is positive mode data and the right panel is negative mode data. The set of ‘iDDA’ means iterative DDA with PMDDA selected precursor ions as the preferred list.

**
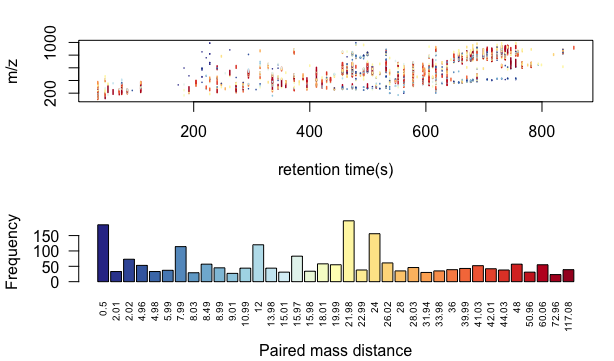
**

Figure S4. high frequency paired mass distances within pseudo spectra in positive mode and their distribution across MS1 data.

**
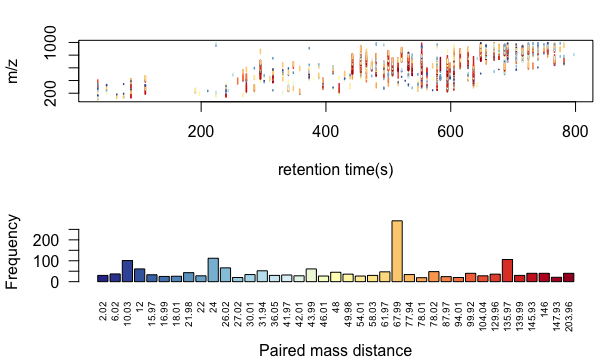
**

Figure S5. high frequency paired mass distances within pseudo spectra and their distribution across MS1 data in negative mode.

**
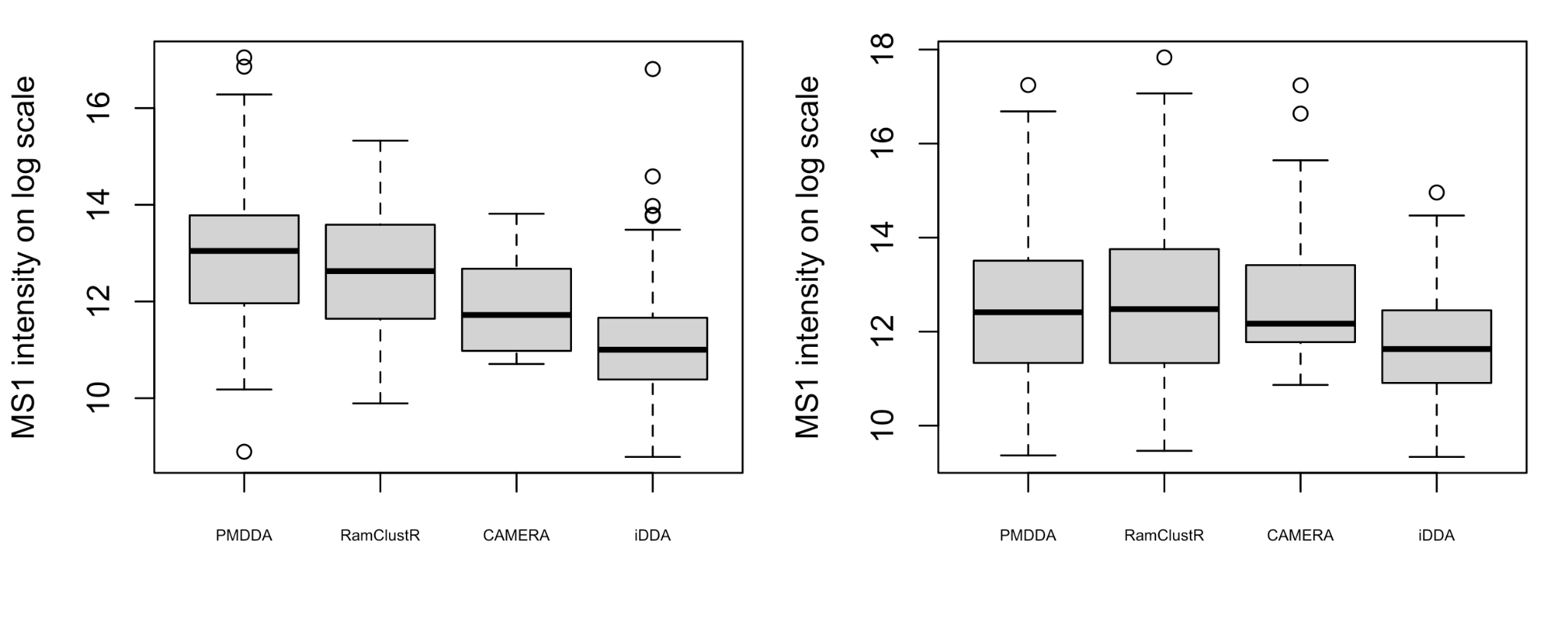
**

Figure S6. Boxplot (left for positive and right for negative) for the full scan peaks intensity of unique precursor ions found in each MS2 spectra from different methods on log scale.
